# Silk Fibroin as a Functional Biomaterial for Drug and Gene Delivery

**DOI:** 10.3390/pharmaceutics11100494

**Published:** 2019-09-26

**Authors:** Mhd Anas Tomeh, Roja Hadianamrei, Xiubo Zhao

**Affiliations:** 1 Department of Chemical and Biological Engineering, University of Sheffield, Sheffield S1 3JD, UK; matomeh1@sheffield.ac.uk (M.A.T.); rhadianamrei1@sheffield.ac.uk (R.H.); 2 School of Pharmaceutical Engineering and Life Science, Changzhou University, Changzhou 213164, China

**Keywords:** silk fibroin, drug delivery, gene delivery, controlled release, bioconjugation

## Abstract

Silk is a natural polymer with unique physicochemical and mechanical properties which makes it a desirable biomaterial for biomedical and pharmaceutical applications. Silk fibroin (SF) has been widely used for preparation of drug delivery systems due to its biocompatibility, controllable degradability and tunable drug release properties. SF-based drug delivery systems can encapsulate and stabilize various small molecule drugs as well as large biological drugs such as proteins and DNA to enhance their shelf lives and control the release to enhance their circulation time in the blood and thus the duration of action. Understanding the properties of SF and the potential ways of manipulating its structure to modify its physicochemical and mechanical properties allows for preparation of modulated drug delivery systems with desirable efficacies. This review will discuss the properties of SF material and summarize the recent advances of SF-based drug and gene delivery systems. Furthermore, conjugation of the SF to other biomolecules or polymers for tissue-specific drug delivery will also be discussed.

## 1. Introduction

**Figure 1:**
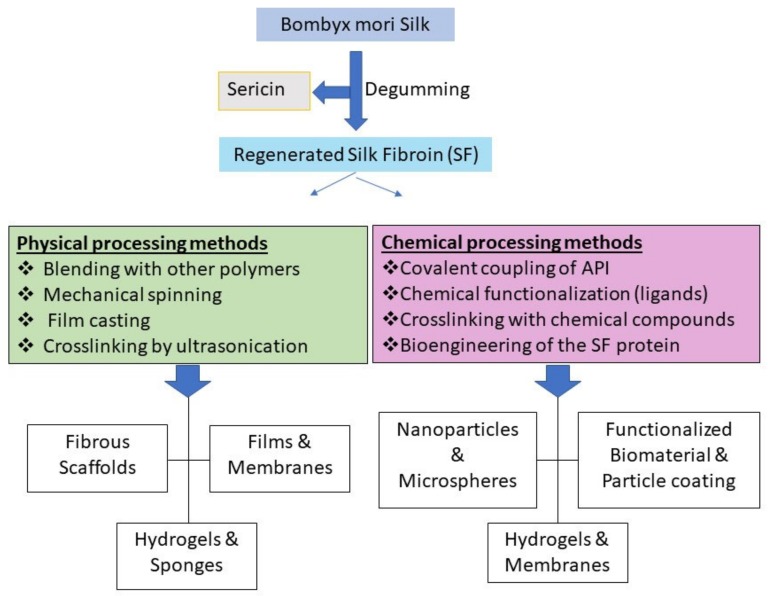
A diverse set of physical assemblies and chemical methods for preparing a variety of silk fibroin (SF) formats for pharmaceutical and biomedical applications.

Polymeric drug delivery systems have emerged as a new efficient alternative to the conventional formulations to provide a reservoir to the active pharmaceutical ingredients (APIs), improve their physicochemical properties, and overcome some of the major challenges in drug delivery including specific targeting, intracellular transport, and biocompatibility in order to improve the treatment efficiency and life quality of patients [1,2,3,4]. An ideal drug delivery system should stabilize the loaded API, allow for modulating its release kinetics and minimize its adverse effects by tissue-specific targeting, especially in the case of highly toxic drugs such as anticancer agents. Silk has been known as a valuable natural material for the fabric industry for centuries, but in the past decades it has attracted immense attention as a promising biopolymer for biomedical and pharmaceutical applications [5,6,7]. Silk protein possesses a unique combination of properties which is rare among natural polymers. It also enjoys desirable characteristics such as mild aqueous possessing conditions, high biocompatibility and biodegradability, and the ability to enhance the stability of the loaded APIs (e.g., proteins, pDNA, and small molecule drugs) [4,5,8,9]. Moreover, silk fibroin (SF) solution can be processed by various methods to produce different types of delivery systems including hydrogels, films, scaffolds, microspheres, and nanoparticles [10]. SF exists in three different structural forms: Silk I, Silk II and Silk III. Silk I exists in water-soluble form and consists of a high percentage of α-helix domains in addition to random coils [11]. Contrarily, Silk II has mainly β-sheet structure and is more stable and water-insoluble, while Silk III prevails at the water/air interface [12]. The transformation from Silk I to Silk II can be tuned by different methods including organic solvent treatment, physical shear, electromagnetic fields, or chemical processing [13,14]. These properties can be utilized in the pharmaceutical industry for producing micro- and nano particles and nano-fibrils or for coating other pharmaceutical preparations such as liposomes [15,16]. Moreover, the availability of carboxyl and amino groups in the SF allows for bio-functionalization with various biomolecules or ligands which could be used for targeted drug delivery [17]. The two main strategies for functionalizing silk protein are chemical conjugation and genetic modification of silk by chainging the amino acid composition or adding a fragment to obtain a specific function [18]. A large proportion of drug formulations including the vast majority of anticancer drug formulations are prepared for parenteral administration, resulting in direct contact with the blood components. Thus, the drug carriers used in such formulations should not induce any haematological toxicity or immune responses [3], which necessitates the use of biocompatible polymers in the formulation. Furthermore, designing delivery systems for biological drugs such as vaccines and antibodies requires maintaining their physical stability as well as their biological activity, which is more crucial for the controlled release systems [19]. This is mainly due to the higher sensitivity of the biological compounds, especially the protein-based therapeutics, to many of the processing conditions throughout the delivery system preparation compared to small molecule drugs, which limits the processing strategies [20,21]. Hence, loading the biological therapeutics into a compatible polymer can increase their stability and consequently their half-life [22]. Moreover, incorporating the APIs into a natural biocompatible protein such as SF has multiple advantages e.g., preserving the API [23], improving the mechanical properties of the formulation [6], modifying drug release kinetics [24,25,26,27], enhancing cell adhesion [16], and compatibility with blood components [25]. The versatility of SF protein processing and formulating methods allows the preparation of a wide range of drug carriers with different sizes and morphologies using unmodified or engineered SF (Figure 1). Unmodified SF carriers have been used to deliver various anticancer drugs such as doxorubicin [28], paclitaxel [29], curcumin [9,30], and cisplatin [31]. In this review, the main strategies for obtaining different SF-based drug delivery systems and the recent methods for generating functionalized SF for controlled or targeted drug and gene delivery will be discussed.

## 2. Physiochemical Properties of Silk Fibroin

SF possesses a unique combination of mechanical and biological properties and exhibits special features of both synthetic and natural polymers [32,33]. Typically, silk represents softness in the clothing industry, but it is considered one of the most robust natural biomaterials due to its tensile strength and modulus [34]. This feature is important for the polymers involved in bone tissue regeneration as mechanical performance of the polymer is of utmost importance for such applications [35]. SF demonstrates excellent stability under high thermal stress (higher than 250 °C) [36].

### 2.1. Biocompatibility

SF is a biocompatible material that has been officially recognized by the Food and Drug Administration (FDA) for the development of a plethora of nanotechnological tools [37]. The biocompatibility of silk has been studied extensively over the past two decades. The majority of the studies have reported excellent biocompatibility and relatively lower immunogenic response in comparison to other common degradable biological polymers in the pharmaceutical industry such as polylactide (PLA), poly(lactic-*co*-glycolic acid) (PLGA) and collagen [22,38,39]. Cytocompatibility studies on SF formats revealed high compatibility with different cell lines including hepatocytes, osteoblasts, fibroblasts, endothelial cells and mesenchymal stem cells (MSCs) [40,41]. During SF processing, organic solvents such as methanol and hexafluoroisopropanol (HFIP) are used to crosslink SF via inducing structural transformation (α helix to β sheet), which has been found responsible for the inflammatory potential of SF formulations [42]. However, mild processing conditions that avoid the use of organic solvents have been used to avoid these inflammatory responses [43]. An evaluation of the levels of lymphocyte activating factor IL-1β and inflammatory cyclooxygenase-2 (COX-2) gene expression in relation to SF stimulation did not show any significant differences from those of collagen or PLA, indicating very low immunogenicity [44]. Another study has evaluated the biocompatibility of scaffolds consisting of a combination of calcium polyphosphate (CPP) and SF used for the reconstruction of cartilage and bone defects [45]. The results showed a tangible increase in tissue biocompatibility and osteogenicity of SF-CPP scaffolds in comparison to CPP scaffolds [45].

### 2.2. Mechanical Properties

Mechanical stiffness is a key property of SF-based formulations for pharmaceutical and biomedical applications. The SF material used in tissue engineering, for example, must match the stiffness of the targeted tissue. The stiffness can also affect the stability and degradability of the SF polymer [46]. Many polymers that have been used in drug delivery devices, such as PLGA and collagen, lack sufficient mechanical strength. A common strategy to enhance the mechanical strength of the biopolymers such as collagen is crosslinking. However, the crosslinking reaction could result in undesirable consequences such as cellular toxicity and immunogenicity [47]. SF possesses robust β-sheet structure which provides excellent mechanical properties without the need for any harsh crosslinking procedures. Based on the β-sheet content, SF can transform into different formats including liquid, hydrogels, or scaffolds [5]. Measurements of mechanical strength are usually obtained from Young’s modulus using nanoindentation techniques [48]. SF exhibits high tensile strength and resistance to compressive force making it a very suitable material for drug delivery and tissue engineering [49]. Moreover, the removal of sericin during the degumming process results in a 50% increase in tensile strength [50] which makes SF more stable during physical pharmaceutical processing.

### 2.3. Stability

The stability of the polymeric materials is one of the most important factors in the production of pharmaceutical formulations. Although biopolymers are preferred to their synthetic counterparts for clinical applications due to their biocompatibility and biodegradability, they must also meet certain stability standards to be considered for utilisation in the pharmaceutical industry. One of the common stability problems of pure SF solution is aggregation or gelation during long-term storage. SF is available in soluble form (with high content of α- helix and random coil) and insoluble form (with high content of β-sheet). Depending on the pharmaceutical preparation, either form should be used and maintained. Storing the soluble SF in highly humid conditions results in a transformation from α-helix and random coil to β-sheet which could lead to gelation and a decrease in the stability of the SF solution [51,52]. SF shows excellent stability under thermal stress compared to other proteins. The best indicator for protein thermal stability is the glass transition temperature (*T*g) which in the case of SF is affected by its β-sheet content. The *Tg* of SF films is approximately 175 °C and the protein remains stable up to 250 °C which is desirable for formulation processing. On the other hand, *T*g of the frozen SF solution can go to −34 °C [53] which is also advantageous in low temperature pharmaceutical processing. Furthermore, the degree of crystallinity and porosity of SF films are also affected by *Tg* [54] The increase in β-sheet content of SF causes a transformation from Silk Ι to Silk ΙΙ, refelected by a significant change in *Tg* which changes the degree of crystallinity. Stability of the SF in physiological fluids is another important issue for its biomedical applications. SF could be protected from enzymatic degradation within the body by coating with polymers such as polyethylene glycol (PEG) in order to improve the delivery of the associated drugs to the site of action.

### 2.4. Degradability

**Figure 2:**
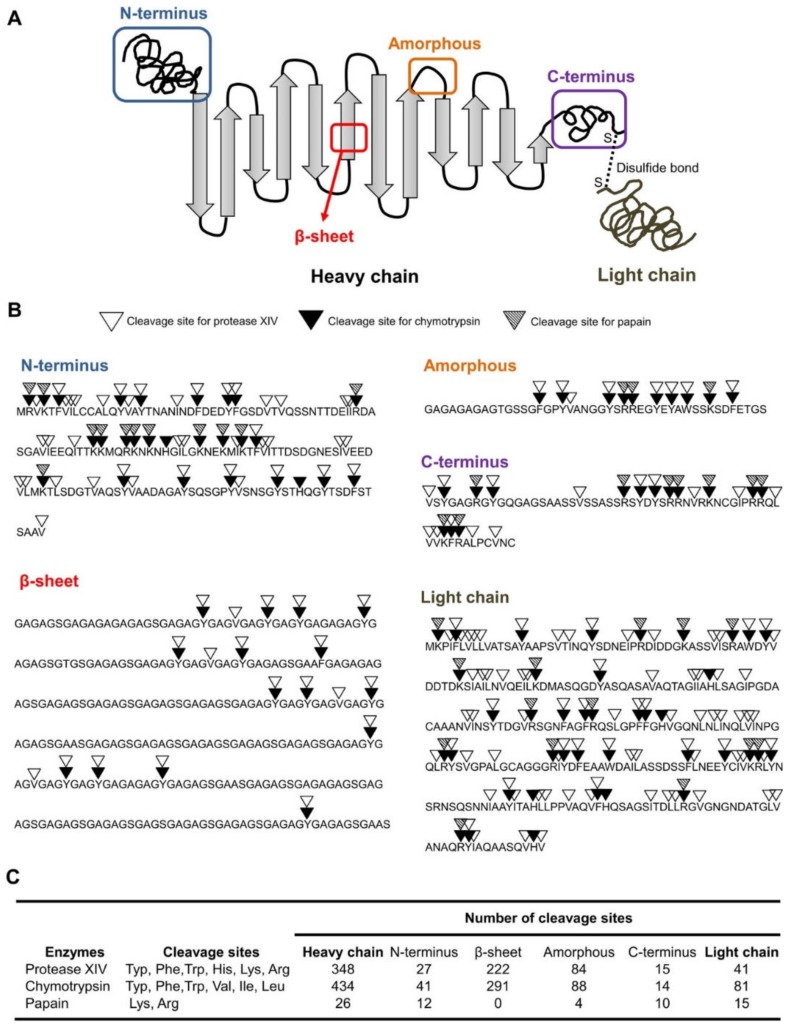
Two-dimensional schematic illustration of silk structure including the heavy chain (i.e., *N*-terminus, crystalline β-sheets, amorphous, and C-terminus) and light chain (**A**). Enzymatic specificities of proteolytic enzymes for the silk sequences (**B**). Number of cleavage sites of proteolytic enzymes on different silk domains (**C**). Reprinted from [55] with permission from American Chemical Society [2018].

Degradability is an important property of biological materials. Although biodegradability is a main advantage of SF in clinical applications, this property makes pure SF particles liable to proteolytic enzymes. The degradation rate of SF can be regulated by modifying the molecular weight, the degree of crystallinity, morphological features, or crosslinking [56]. However, the degree of crystallinity and crosslinking are not the only approaches to stabilize SF against degradation. For example, an in vitro enzymatic degradation experiment revealed that SF sheets slightly transformed from Silk II to Silk I crystalline structure when exposed to collagenase IA. However, when protease XIV was used, the majority of the SF sheets transformed to Silk I leading to a higher degree of crystallinity. Although the degradation time was 15 days in both cases, the degradation rate was significantly lower for protease XIV compared to collagenase IA [57]. Another study reported a predictable loss of mechanical integrity due to SF degradation [58]. Incubation with protease led to an exponential decrease in the SF filament diameter to 66% of the initial diameter after 10 weeks. Gel electrophoresis indicated a decreasing amount of the silk 25 kDa light chain and a shift in the molecular weight of the heavy chain with increasing incubation time with protease XIV [58]. The enzymatic degradation behavior of SF was studied by Wongpinyochit et al. [55] using three different proteases (papain, chymotrypsin, and protease XIV) over a period of 20 days. As shown in Figure 2, the cleavage sites vary from one protease to another, leading to variations in degradation rate. The degradation rate was higher in the presence of chymotrypsin compared to papain and protease XIV but the latter two did not have any significant difference. The degradation rate does not only rely on cleavage sites but also on the enzyme accessibility, SF format, and the secondary structure of the SF [55]. A previous in vivo degradation study in rats found that SF degredation is also related to the phagocytic activity of the cells (fibroblasts) and the presence of sericin with SF [59]. The raw silk (SF and Sericin) has a higher degradation rate than pure SF because sericin promotes degredation by the cells [59].

## 3. SF-Based Drug Delivery Systems

Delivery of APIs in sustained and controlled release forms is important for many clinical applications. Selection of the particle size, composition, and other features depends on the type of the delivery system and the route of administration. Moreover, using biocompatible and mechanically durable polymers with mild fabrication and processing conditions in such delivery systems is advantageous for preserving the bioactivity of the loaded APIs. As discussed earlier, SF meets all these requirements which makes it a promising candidate for drug delivery [5,60]. SF-based drug delivery systems can be fabricated by different methods, each resulting in a delivery system with unique properties such as modified release kinetics, stability, and other features which could be of benefit in various applications. Various types of SF-based drug delivery systems have been designed including hydrogels, films, micro- and nanoparticles, nanofibers, lyophilized sponges as well as SF-coated polymeric particles. In the following section, some of the most widely studied SF-based drug delivery systems are reviewed.

### 3.1. Hydrogels

SF aqueous solution was used to generate hydrogels by different methods. The transition from solution to gel can be triggered by physiochemical or chemical processes using natural polymers or synthetic reagents [61]. The physicochemical processes include shearing (spinning), water exclusion via evaporation or osmotic stress, electric field, and heating [62]. The gel form is stabilized because of thermodynamically stable β-sheets which result in a stable gel form in physiological conditions unless extensively degraded by enzymes or oxidative reactions [62]. One recent study used curcumin-loaded gel scaffolds prepared by electrogelation for wound healing [63]. The prepared gel formulation not only improved protein adsorption and sustained the release of curcumin, but also enhanced bacterial growth inhibition by 6-fold against *S. aureus* [63]. Since protein adsorption on substrates is a key factor for cell growth and proliferation, SF gel scaffolds can serve in wound healing by promoting cell proliferation. Sundarakrishnan et al. [64], adapted a chemical approach using horseradish peroxidase (HRP) and hydrogen peroxide to prepare SF hydrogels that were subsequently crosslinked with di-tyrosinase, and loaded with phenol red in order to develop a self-reporting pH system for in vitro environment [64]. Addition of phenol red during di-tyrosine crosslinking resulted in stable entrapment of phenol red within SF hydrogel network due to covalent interactions between phenol red and tyrosine and also prevented leaking [64].

### 3.2. Silk Films

Film preparation from SF has attracted more attention recently due to its huge potential as a biomaterial in pharmaceutical formulations and tissue engineering [65]. SF films can be simply prepared by casting an aqueous SF solution [66]. However, there are other reported SF film preparation techniques such as vertical deposition [67], spin coating [68], centrifugal casting [65], and spin assisted layer-by-layer assembly [69]. Terada et al. [68] investigated the behavior of spin-coated SF films treated with different ethanol concentrations. Alcohol concentrations of 80% or less resulted in a jelly-like hydrogel layer while treatment with more than 90% alcohol provided a rigid film surface. This change in morphology affected the attachment of the fibroblast cells to the SF films. Fibroblasts aggregated on the rigid surface rather than attaching individually to the hydrogel surface [68]. Another study found that blending SF with other polymers such as sodium alginate (SA) before casting the film results in a miscible and transparent film and also induces a structural change in SF [70]. Manipulating the SF/SA blending ratio shifted the SF conformation to the higher β-sheet content. Moreover, mixing SA with SF enhanced water permeability, swelling capacity and tensile strength of SF films [70]. Hence, SF/SA blend can provide unique tunable chataristics that can be benificial in pharmaceutical applications.

### 3.3. Silk Particles

**Figure 3:**
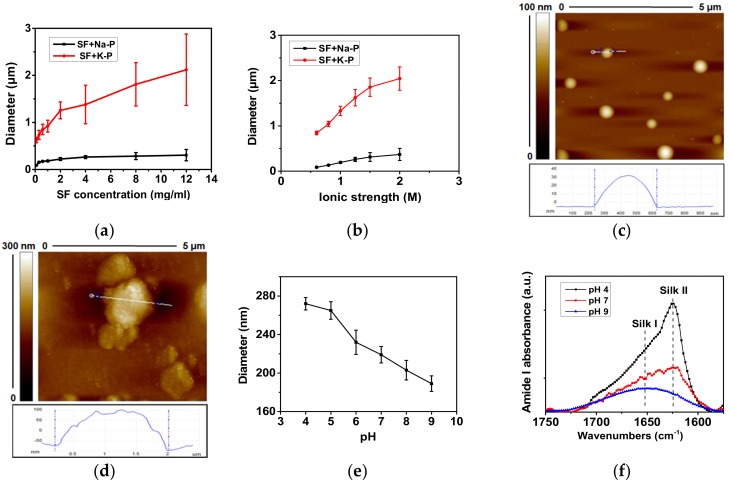
Effects of SF concentration, salt, ionic strength and solution pH to the particle size and protein secondary structure. (**a**) Particle diameter as a function of SF concentration when adding SF solutions (concentration from 0.1–12 mg/mL) to sodium phosphate (Na-P) and potassium phosphate (K-P) solutions (both at ionic strength 1.25 M, pH 8) at the volume ratio of 1:5. (**b**) Diameter of SF particles fabricated with K-P or Na-P as a function of their ionic strength. The SF concentration was fixed at 5 mg/mL. (c-d) AFM images of particles fabricated by adding SF solution (12 mg/mL) in to sodium phosphate (**c**) and potassium phosphate (**d**). Both solutions are at the ionic strength of 1.25 M and pH 8. (**e**) Diameter of SF particles fabricated with Na-P as a function of the Na-P solution pH. (**f**) FTIR spectra of particles produced by 1.25 M sodium phosphate at different pH values. It was found that the use of sodium phosphate, lower ionic strength and higher pH of solution produces smaller SF particles. Reprinted from Reference [43]; with permission from American Chemical Society [2017].

As discussed earlier, the are an increasing number of SF-based systems that have been used for encapsulating APIs and achieving modulated drug delivery among which nanoparticle delivery systems have been studied the most, especially for anticancer drugs. One example of such systems is the lysosomotropic SF nanoparticles designed by Seib et al. [28] for pH-dependent release of the anticancer drug doxorubicin in order to overcome drug resistance. SF nanoparticles are largely employed for controlled release of the loaded drug at the site of action. SF nanoparticles can be fabricated by various methods, for example polyvinyl alcohol (PVA) blends, which are used for fabricating SF spheres with controllable sizes and shapes [71] (Table 1). The determinant factors for drug distribution and encapsulation efficiency in such systems are their charge and lipophilicity. Modifying these factors results in different drug release profiles [72]. Furthermore, addition of PVA results in a tangible improvement in the morphology of the SF-particles [73]. One of the popular methods for fabrication SF particles is the salting-out method. For example, Lammel et al. [74] produced SF particles with controllable sizes ranging from 500 nm to 2 µm using potassium phosphate as the salting out agent. The β-sheet structure and zeta potential of the SF particles were affected by the pH of the potassium phosphate solution [66,74]. In another study conducted by Tian et al. [75] SF nanoparticles were prepared using the salting out method, loaded with a combination of doxorubicin and Fe_3_O_4_ magnetic nanoparticles and driven to the target tissue using an external magnetic field to achieve tissue-specific targeted delivery [75]. It was also found that the entrapment efficiency of doxorubicin can be tuned by changing the concentration of Fe_3_O_4_ in the formulation [75]. However, the size of SF particles produced by potassium phosphate was over 500 nm, which is not ideal for drug delivery. Recently, Song et al. [43] produced magnetic SF nanoparticles (MSNPs) with size range of 90–350 nm by using sodium phosphate. The size and morphology of the MSNPs were governed by the SF concentration, the ionic strength and pH of the salting-out agent (Figure 3) [43]. Compared to potassium phosphate, sodium phosphate produced smaller particles and the size did not increase significantly with increasing SF concentration and ionic strength, providing a promising method to produce smaller particles with high concentration for drug delivery. The size of the particles can be further reduced by increasing the pH of the salting-out agent (Figure 3). Although salting-out and PVA methods are preferred over other methods due to their simplicity and low toxicity, purifying the SF nanoparticles from excess polymers or salting-out agent is required. Therefore, Mitropoulos et al. [76] managed to prepare SF particles with spherical shape using a co-flow capillary device with PVA as the continuous phase and silk solution as the discrete phase. This device allows for generation of SF spheres (2 µm in size) without the requirement for any further purification steps. Moreover, the diameter of the spheres can be simply adjusted by changing the concentration of the polymers, the flow rate, and the molecular weight of the selected polymer [76]. However, the size of the particles produced is not within the desired size range for drug delivery. A more recent study on the microfluidics has used a microfluidic set up (nano-assembler) to produce smaller sizes of SF particles (150–300 nm) by a desolvation method [77] (Table 1). It was found that the characteristics of the SF nanoparticles are controlled by two main factors: Flow rate and flow rate ratio [77]. The use of microfluidic instrument enabled rapid, reproducible and controlled production of SF nanoparticles with desirable sizes for drug delivery. However, solvent residues within the particles and the cost of the equipment should also be taken into account. The properties of the SF particles can also be manuplated by blending with other polymers. For example, Song et al. [9] have recently produced SF nanoparticles blended with different amounts of polyethyleneimine (PEI). The size of the SF nanoparticles was found to increase with increasing SF percentage (Figure 3), while the zeta potential of the particles decreased with increasing SF amount. This allows to fine tune the drug delivery through controlling the size and zeta potential of the particles.

## 4. Applications of Silk Fibroin for Drug and Gene Delivery

Silk has been used as a carrier for delivery of a wide range of therapeutic agents including small molecule drugs [3], biological drugs [84], and genes [85]. For each class of therapeutic agents, different formulations have been designed using various silk processing technologies [86]. One of the main criteria of the SF-based delivery systems is to stabilize the loaded API and manipulate its circulation time to achieve the required therapeutic effect. In addition, the designed formulations are usually optimized to obtain a particular application in drug delivery including stabilising the loaded drug, controlling drug release, and improving cell adhesion [16]. In the following section, an insight into SF applications in drug and gene delivery will be provided a summary of which is presented in Table 2 and Table 3.

### 4.1. Drug and Gene Stabilization by SF

One of the main goals of incorporating active ingredients such as small molecules or peptides into SF-based carriers is to stabilise them by different mechanisms including adsorption, covalent interaction, and/or entrapment [87]. Without a stable interaction between the drug and the SF-based carrier to maintain the drug activity, sustained drug release cannot be achieved. Aside from a few exceptions such as growth factors, the majority of the stabilisation approaches rely on entrapping the drug within the SF-matrix or SF-particles in an equally distributed manner [22]. SF-based biomaterials are generally stable to changes in temperature [23], humidity [88], and pH [89]. Therefore, they have been widely studied for enhancing the stability of other materials, for example, encapsulation of antibiotics such as erythromycin, which has very low stability in water. However, porous SF sponges managed to sustain its release and maintain its antimicrobial activity against *Staphilococcus Aureus* for up to 31 days at 37 °C [3]. SF films have also been used for stabilisation of biological compounds. For example, enhanced stability of horseradish peroxidase (HRP) when loaded on SF films or mixed with SF solutions has been reported. The enzymatic activity of SF-loaded HRP was increased by 30–40%, while its half-life showed a tremendous increase from 2 h to 25 days at ambient conditions in comparison to free HRP [90]. A greater improvement in enzymatic activity (80%) was observed in glucose oxidase (GOx) when loaded on SF films [91]. Moreover, SF-loaded GOx demonstrated enhanced thermal and pH stability [92]. Topical application of SF lyogels (gel system in which the pores are filled with both organic and non-organic solvents) containing hydrocortisone in a mouse model of atopic eczema resulted in decreased expression of IgE and enhanced the efficacy of hydrocortisone compared to the commercially available hydrocortisone cream [93]. Moreover, SF lyogels have also been used for stabilising monoclonal antibodies. The lyogels achieved sustained release of IgG1 over 160 days and the release rate was found to be inversely proportional to the SF concentration [19]. In addition to drug stabilisation, SF has also been investigated for DNA preservation in order to protect the DNA from the potential destabilising conditions such as temperature and UV radiation. In a recent study porous cellulose paper was coated with SF and used to preserve the DNA extracted from human dermal fibroblast cells [23]. The results showed that the DNA integrity was maintained for 40 days following 10 h of UV radiation at relatively high temperature (37–40 °C) [23].

### 4.2. Controlled Drug Release

Controlled release drug delivery systems are aimed at releasing the encapsulated API in specified amounts over a specified period of time. One application of such systems is sustained drug release to maintain the therapeutic concentrations of the drug in the blood or site of action for a longer duration which is of great importance for the treatment of chronic diseases. Moreover, sustained drug release reduces the administration frequency and the adverse drug reactions which results in increased patient compliance [22]. Most of the currently available controlled release formulations in the market are composed of synthetic polymers such as PEG and PLGA because they provide desirable pharmacokinetic and pharmacodynamic properties [94]. Although PLGA is approved by FDA as a safe ingredient in pharmaceutical products, the processing requirements might restrict its utilisation in certain controlled release formulations. Therefore, more recently, natural polymers such as SF which offer tunable sustained release kinetics and stabilization of the loaded APIs have gained more attention for use in controlled drug release systems.

**Figure 4:**
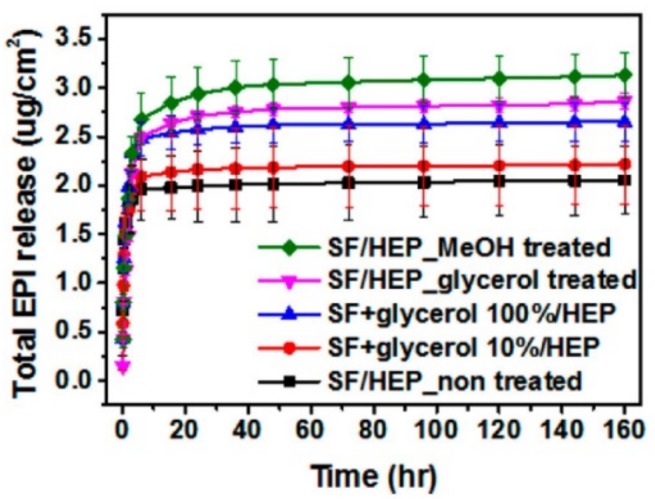
Total epirubicin (EPI) release profile from SF nanofilm depending on the ratio of the added glycerol and the solvent treatment. Reprinted from Reference [7] with permission from the American Chemical Society.

One of the unique properties of SF is its ability to undergo diverse structural transformations at the molecular level. The most investigated structural transformation in SF is the change in the ratio of α-helix to β-sheet content. For example, the permeability and release kinetics of the SF films are affected by the percentage of β-sheet structure [7]. The mechanism of controlled release from SF films was studied previously by Hines and Kaplan using different models [95]. The release kinetics of FITC-dextran from methanol-treated and untreated SF films was evaluated as a function of molecular weight of FITC-dextran. The methanol-treated films maintained higher percentage of the loaded FITC-dextran compared to the untreated films which was directly proportional to the molecular weight of FITC-Dextran [95]. In a more recent study, the release profile of the anticancer drug epirubicin from five Heparin-SF films (HEP-SF) treated with methanol (MeOH) or glycerol was investigated and it was found that using different ratios of glycerol in the HEP-SF nanofilm formulation affects the β-sheet content of the nanofilm leading to a modification in the release profile of epirubicin from the nanofilm (Figure 4). This mechanism-causal relationship between SF conformation and release profile also influenced the degree of degradation [7].

In a novel study conducted by Yavuz et al. [27] SF was formulated into insertable discs that can encapsulate either IgG antibody or human immunodeficiency virus (HIV) inhibitor 5P12-RANTES. Three different formulations were prepared by SF layering, water vapor annealing, and methanol treatment. These formulations managed to stabilize the protein cargo and to modify its release profile. High concentrations of IgG were released in a relatively short time from the formulation treated with methanol due to the highly porous structure in comparison to the other two formulations that demonstrated a slower and more controlled release. In the case of 5P12-RANTES, the water vapor annealing showed a sustained release for 31 days and this released protein could inhibit HIV infection in both blood and human colorectal tissue [27].

**Figure 5:**
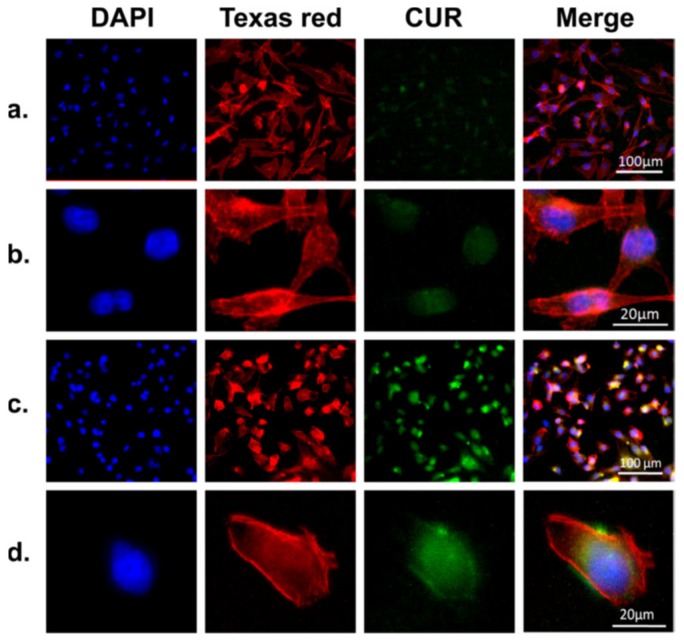
Representative microscopic images of MDA-MB-231 cells incubated with free curcumin ((**a**) and (**b**), curcumin amount (10 μg/mL), equivalent to the curcumin amount in CMSPs (curcumin loaded magnetic SF core−shell nanoparticles) and CMSPs ((**c**) and (**d**), 30 μg/mL) for 4 h. The cell nucleus and cytoskeleton were stained with DAPI (blue) and Texas red (red); all images were taken with an AF6000 microscope (Leica). Comparing the images in (**a**) and (b) to (**c**) and (**d**), it can be seen that CMSPs significantly improve the cellular uptake of the curcumin. Reprinted from Reference [43] with permission from the American Chemical Society [2017].

Controlled release from SF nanoparticles and microspheres has been studied extensively in the past decade. In an attempt to control SF particle features, a recent study conducted by Song et al. [43] demonstrated pH-controlled release of curcumin from SF nanoparticles for up to 20 days with lower pH promoting the release. Moreover, the SF nanoparticles had higher cellular uptake and induced significantly higher growth inhibitory effect in MDA-MB-231 cells compared to curcumin solution (Figure 5).

Another study developed SF microspheres (2 µm) using DOPC (1,2-dioleoyl-*sn*-glycero-3-phosphocholine) lipid vesicles as a templates [96]. The physically cross-linked β-sheet structure of SF and the residual DOPC in the microspheres played key roles in controlling the release of loaded enzyme (HRP) [96].

In addition to the controlled release systems composed of SF as their main component, SF has also been used as coating to modify the release kinetics of drug delivery systems made of other polymers.

**Figure 6:**
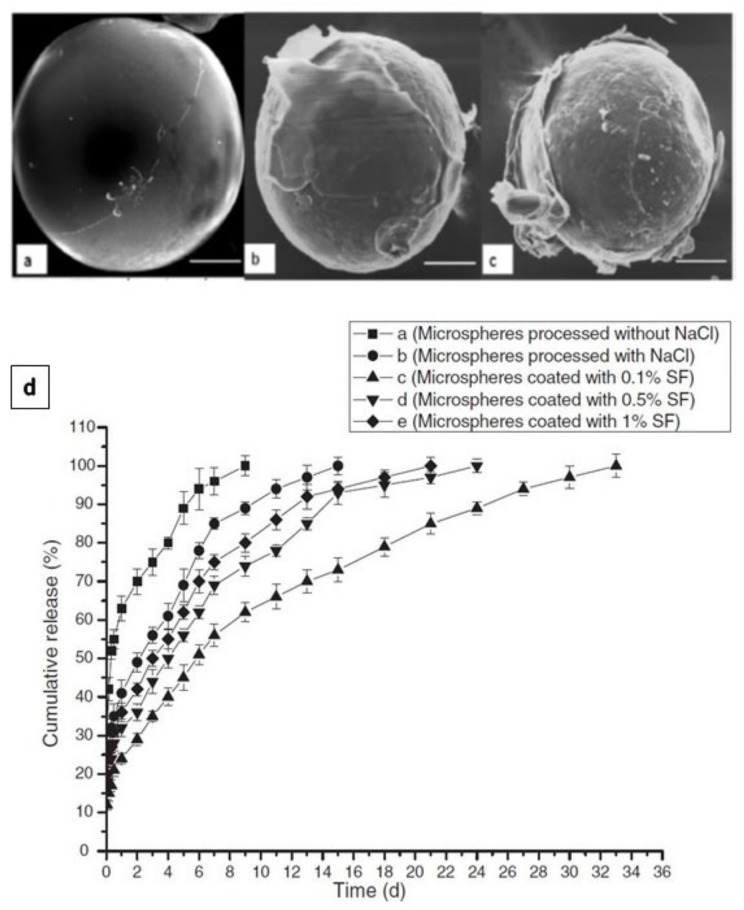
Scanning electron microscope (SEM)images of microspheres coated with 0.1% SF (**A**), 0.5% SF (**B**) and 1% SF (**C**). Microsphere coated with 0.1% SF showed a smooth surface without defects or cracks. Microspheres coated with 0.5% and 1% SF showed poor coating comprised of numerous surface defects and cracks in the film structure (bar size 50 mm). In vitro release of vancomycin from microspheres coated with different concentration of SF (**D***)*. Reprinted from [98] with permission from Taylor & Francis Online [2011].

SF, as a biopolymer, can be processed in aqueous conditions and crosslinked by different methods. Therefore, SF solution has been utilised for single or multilayer coating of different pharmaceutical preparations. In a study conducted by Pritchard et al. [97], the adenosine release from SF encapsulated powder reservoirs was evaluated as a function of reservoir coating thickness. The coating thickness was varied by changing the concentration of the silk coating solution and the number of coating layers applied. Increasing the coating thickness or the crystallinity of the SF delayed adenosine burst, decreased average release rate, and increased the duration of release [97]. Eliminating infections by releasing antibiotics such as vancomycin from biodegradable microspheres is a very effective strategy. However, maintaining the antibiotic concentration within the therapeutic window over the required treatment time remains a challenge [98]. A recent study has addressed this challenge by coating vancomycin-loaded poly(*ε*-caprolactone) (PCL) microspheres with SF to reduce the burst release of vancomycin [98]. The PCL microspheres were prepared by double emulsion (W_1_/O/W_2_) solvent evaporation/extraction process and the coating was performed by suspending the microspheres in the SF coating solutions (0.1%, 0.5% or 1%). Methanol was used to induce SF transformation from α-helix to β-sheet [98]. The microspheres coated with 0.1% SF showed smooth surface and presented a better release profile. By increasing the SF concentration, more cracks and defects were detected (Figure 6) [98].

Many studies have focused on improving the efficiency of the current nanoformulations and increasing the bioavailability of the loaded drugs by using adhesive excipients. For example, ocular drug delivery is a very challenging task and requires highly optimized formulations due to the unique environment in the eye. Because of the low ocular bioavailability, the frequency of applying the eye drops is usually high which can cause cellular damage at the ocular surface [99]. Increasing the residence time of drug on the eye surface will not only improve the efficiency of the therapy but also reduce the frequency of administration. A study presented by Dong et al. [16] prepared SF-coated liposomes loaded with ibuprofen for ocular delivery. The SF-coated liposomes exhibited better cell adhesion in human corneal epithelial cells (HCEC) compared to the conventional liposomes. Moreover, the drug release and permeation rates could be tuned by adjusting the concentration of the SF [16]. Another study also used SF coating on emodin-loaded liposomes (SF-ELP) to enhance keloids cell adhesion [100]. This study showed a selective targeting of keloids cells in comparison to normal cells which was achieved through interaction between SF and the cell in contrast to ligand targeting which is achieved by binding to specific receptors on the cell surface. SF-coating also limited Brownian motion and increased the probability of the nanoparticles attaching to the cell surface [100].

### 4.3. Gene Delivery

Gene delivery is defined as the introduction of genetic material including DNA and RNA into the targeted cells to regulate the expression of particular genes or direct the synthesis of specific proteins in order to treat disorders caused by dysregulation or malfunction of those proteins [101]. Using plasmid DNA (pDNA) is more common than RNA in gene delivery, especially to cancer cells [38,101]. Despite that more than 2600 clinical trials on gene therapy have been carried out in 38 countries since the early 1990s, none of these therapies have been granted FDA approval [102]. One of the main reasons why the gene-based formulations have failed to find their way to the market is that the majority of these formulations use viruses as gene vectors. Viral vectors usually give rise to concerns regarding systemic toxicity and immune response [38]. Therefore, the recent studies have shifted their focus toward non-viral vectors using biocompatible polymeric formulations. Non-viral gene delivery systems are typically designed using cationic lipids such as DOTAP (1,2-bis(oleoyloxy)-3-(trimethylammonio)propane) or positively charged synthetic polymers such as polyethyleneimine to provide a safer alternative to viral vectors [103]. However, transfection efficiency, target specificity and cytotoxicity of the common non-viral vectors remain obstacles to overcome. In addition to biocompatibility, SF has demonstrated DNase resistance and high transfection efficiency. These properties make SF a preferable polymeric vector for gene delivery [104]. Using genetic engineering, SF can be modified to gain more functions that suit the desired application. For example, modifying silk by adding PLL (poly(L-lysine)) sequences resulted in higher transfection efficiency of pDNA in human embryonic kidney (HEK) cells [105]. A later study has used SF bioengineered with PLL domains to interact with both pDNA and tumor-homing peptide (THP) for targeted pDNA delivery [85]. Even though silk based gene delivery vectors have shown high transfection efficiency and an acceptable degree of specificity [85], higher specificity is required for more effective targeted cancer therapy. An improved silk-based gene delivery system has been developed by adding F3 and Lyp1 peptides to recombinant silk proteins with a relatively high content of THP (25 mol%, 3.4 kDa/13.6 kDa) [103]. F3 peptide is capable of specifically binding to MDA-MB-435 cancer cells while Lyp1 binds specifically to tumor lymphatics and can also induce cell death in MDA-MB-435 cells [106,107,108,109]. The designed system achieved specific delivery of pDNA to the tumorigenic cells [103].

Another approach to capture DNA is using cationic polymers such as polyethyleneimine (PEI) within the silk formulation [9]. Luo et al. [4] developed cationic SF scaffolds by coating PEI on SF scaffold for delivery of vascular endothelial growth factor 165/angiopoietin-1 coexpression plasmid DNA (pDNA & VEGF165–Ang-1). PEI coating converted the surface charge from negative to positive through amidation reaction between the spermine and the carboxyl groups in the side chains of SF. The positive surface charge allowed the SF scaffold to form complexes with pDNA which demonstrated higher transfection efficiency and lower cytotoxicity than PEI/DNA complexes [4]. SF coated PEI/DNA complexes were also used to transfect HEK 293 and human colorectal carcinoma HCT 116 cells, and the system exhibited higher selectivity for HCT 116 compared to HEK 293 [110].

Recently, Song et al. [9] investigated the delivery of oligodeoxynucleotides (ODNs) to MDA-MB-231 breast cancer cells. The addition of SF to the nanoparticle formulation not only increased the cellular uptake of ODN (70%) but also significantly reduced its cytotoxicity (Table 3) [9]. Cell-penetrating peptides (CCPs) have also been used in SF-based gene delivery systems due to their ability to penetrate or destabilize cellular membranes. When designing a non-viral carrier for gene delivery, CCPs are considered among the preferred components to facilitate clathrin-dependent endocytosis of the particles [111,112]. Functionalization of silk protein with ppTG1 which is a CPP increased its transfection efficiency for pDNA in HEK 293 cells compared to unmodified silk (Table 3) [113].

## 5. Modification of SF for Enhanced Delivery

### 5.1. SF Bioconjugates

**Figure 7:**
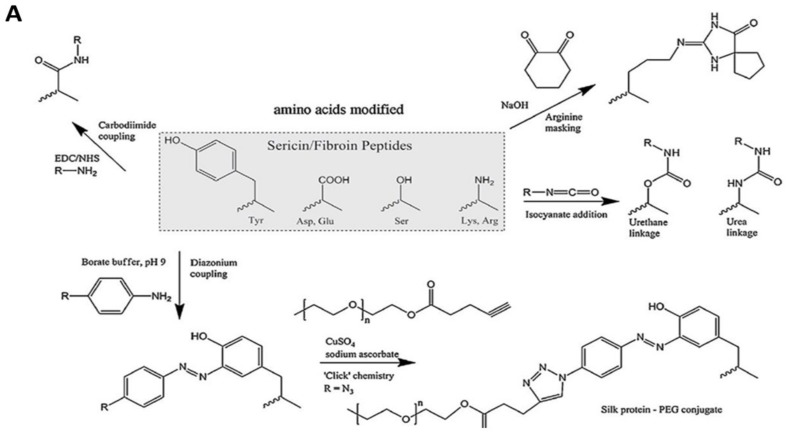

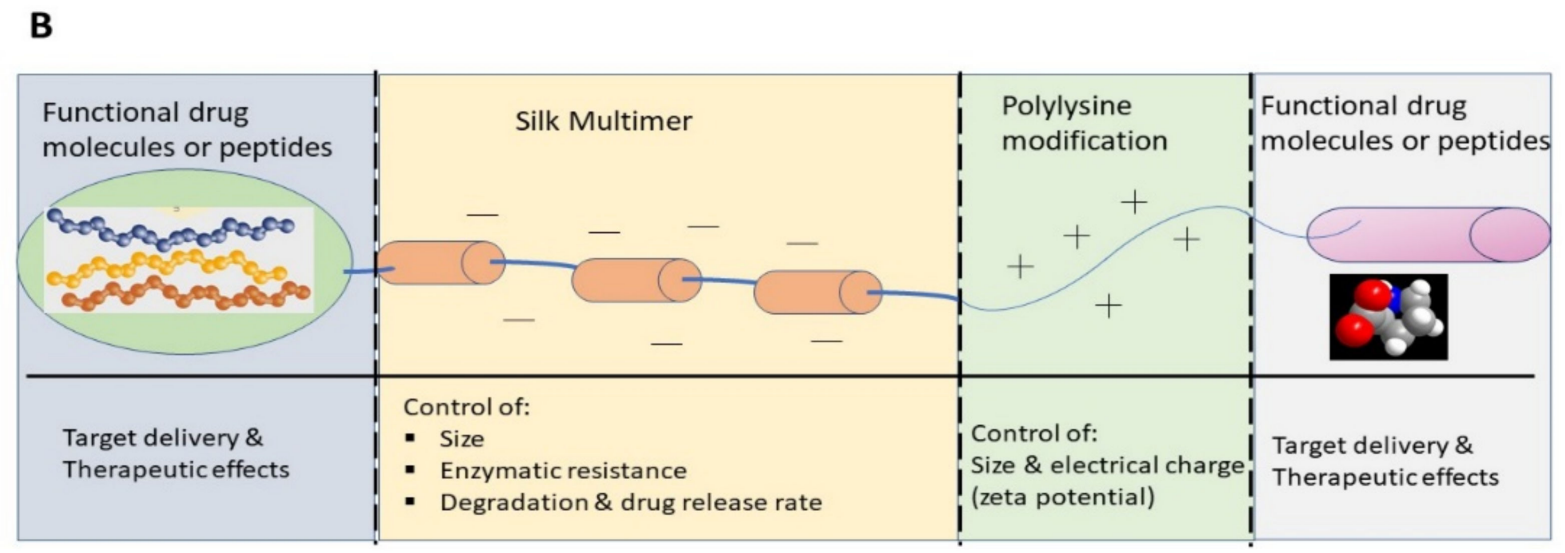
(**A**) Possible routes toward chemical modification of amino acids of silk proteins. Reprinted from [50] with permission from Elsevier. [2014].(**B**) The new properties obtained by SF when functionalised or modified in different positions.

There are many protein-based drugs that have shown a very short half-life in the body. In order to enhance their in vivo stability, an approach has been designed to utilize SF by forming bioconjugates. A covalent bond between the protein or enzyme and SF can be formed by the cross-linking reagents [5]. SF consists of 18 different amino acids among which 10% are polar amino acids such as serine and lysine with hydroxyl and amino groups in their side chains. These functional groups in SF can be covalently conjugated to polar groups in other proteins such as insulin using bifunctional reagent glutaraldehyde [119]. SF-insulin (SF-Ins) bioconjugate not only demonstrated higher in vitro stability than bovine serum albumin-insulin (BSA-Ins) conjugate, but also prolonged the pharmacological activity 3.5 times in comparison to native insulin [119]. Covalent conjugation of growth factor BMP-2 to SF using carbodiimide chemistry preserved BMP-2 activity and also reduced its degradation rate due to reduction in its unfolding rate as well as protecting it from proteases [120]. Immobilization of enzymes on silk particles has also been studied recently to enhance the catalytic efficiency of enzymes by improving enzymatic stability. SF has several active amino groups that have the potential for covalent binding to several enzymes to immobilize them (Figure 7) such as catalase immobilization on SF particles via tyrosinase crosslinking [121]. SF films have been also used to immobilize antibodies such as mouse IgG simply through the conformational transition to fabricate biocompatible biosensors [122]. The immobilization was achieved by slowly drying concentrated SF solution to reach the semisolid state and then blending it with antibody solution before complete drying. It was found that more antibody was immobilized on the surface of SF film by controlling the conformational changes during the drying process in comparison to covalent methods [122]. These results indicate that SF can be functionalized with antibodies with or without crosslinking agents, offering a wide range of biomedical applications.

### 5.2. Functionalization of SF with Ligands

One of the fundamental advantages of nanoparticles is the greater surface area to the volume ratio compared to larger particles. This property is essential for encapsulating the APIs such as anticancer agents and delivering them to the site of action. Moreover, the loaded APIs must be delivered at a proper concentration to cause the required effect on the target cells and minimise the damage to other cells [123]. However, recent studies have found that engineered particles with the optimum size and shape are limited to less than 1% tumor tissue accumulation [124]. Therefore, decorating polymeric nanocarriers with a targeting molecule has emerged as an effective approach to increase the specificity of the nanoparticles for the targeted cell lines [125]. As mentioned earlier SF has several active amino groups (Figure 7) which can be used for binding to other macromolecules [126]. For example, Arg-Gly-Asp (RGD) sequence that acts as a ligand for cell surface integrin receptors can be linked to SF particles to enhance their attachment to the certain cancer cells that overexpress integrins [127]. In a similar fashion, due to the overexpression of folate receptors (FR) in a wide range of tumor cells, modifying the surface of the silk nanoparticles with folate could be used as a tumor-targeting strategy [128]. Folate-conjugated SF particles (SF-FA) were used to enhance targeted delivery of doxorubicin (DOX) to human breast adenocarcinoma cell line (MDA-MB-231) [127]. Folate decoration on silk particle not only increased the retention of the nanoparticles at the tumor site but also promoted cellular uptake of the particles [129]. DOX incorporated in SF-FA nanoparticles demonstrated 3-folds higher cytotoxic activity in comparison to free DOX *in vitro*. Moreover, conjugation of folate to SF nanoparticles changed their cellular uptake mechanism from passive diffusion (free DOX) to endocytosis [126]. Another example of specific targeting using functionalized SF is modification of SF with human epidermal growth factor receptor 2 (Her2) which is overexpressed in 30% of breast carcinomas for targeted drug delivery to breast cancer cells [130]. An alternative functionalization approach involves using tumor-specific ligands such as nucleic acid sequences like CpG-siRNA [131].

## 6. Conclusions

Silk is a versatile biomaterial with great potential for drug and gene delivery applications. SF has been used as a naturally derived biopolymer for development of various types of drug delivery systems including hydrogels, SF films, microparticles and nanoparticles using a variety of fabrication methods. Each of these SF-based systems have shown promising features for different biomedical applications. SF micro- and nanoparticles have been used for delivery of different types of drugs such as curcumin, doxorubicin and ibuprofen as well as pDNA to various types of cells in a time-specific or site-specific manner. SF films have been used for controlled release of drugs such as dextran, epirubicin, and biological agents such as IgG and HIV inhibitor 5P12-RANTES. In addition, they have been used to stabilise biological agents such as horse radish peroxidase (HRP), glucose oxidase, vaccines and monoclonal antibodies in order to enhance their shelf life. Moreover, conjugation of SF to biomolecules such as insulin and BMP-2 has been employed as a strategy to sustain their release and prolong their biological activity. Functionalisation of SF with biological recognition elements such as RGD sequence, folate and Her2 has been used for tissue-specific drug delivery. In addition to the SF-based drug delivery systems, SF has also been used for coating the surface of polymeric microparticles and liposomes in order to modify their release kinetics or enhance their cell adhesion. However, despite all the advances made in the fabrication of SF-based constructs for biomedical and pharmaceutical applications, there is still lack of sufficient studies on the applications of bioengineered or structurally modified silk for tissue-specific targeted drug and gene delivery. Also, modifying the physichochemical and mechanical properties of the SF through miximng it with other natural or synthetic polymers in order to develop tailored SF-based biomaterials is another area to be further explored.

## Figures and Tables

**Table 1:** Preparation teqniques of SF micro- and nanoparticles.

Preparation Technique	Advantages	Disadvantages	Particle Size
**Self-assembly**	Simple and safe procedure Does not require toxic reagents	Sensitive to temperature and vigorous mixing	100–200 nm [11]
**Salting out**	Low cost method The active ingredient can be loaded during the particle formation	Salting out agent residue Relatively high particle size polydispersity	100–350 nm [43] 500 nm–2 µm [74]
**Emulsification**	Controllable particle size Low cost method	Organic solvent or surfactant residues	170 nm [78]
**Desolvation**	Simple and quick method Small particle size Reproduceable technique	Particle aggregation Organic solvent residue	35–170 nm [79]
**Electrospraying**	High purity particles Very good monodispersity	Requires additional step to insolubilize SF	59–80 nm [80] 600–1800 nm [72]
**Microfluidic methods**	Rapid procedure Mild operation conditions Controllable particle yield and particle size	Relatively expensive Residual salting agent or organic solvents	150–300 nm [77]
**Capillary microdot**	Simple procedure	Organic solvents residue	25–140 nm [81]
**Freeze drying**	Porous particles	Large particle size	490–940 µm [17]
**Supercritical fluids**	High drug loading	Expensive technique Not easy to operate Requires additional step to insolubilize SF	50–100 nm [82]
**PVA Blending method**	Time and energy efficient No use of organic solvent	PVA residue	5–10 µm [71] 300–400 nm [71]
**Nano-imprinting and inject printing**	Tuneable dimensions of different nanostructures	Complicated method Not easy to scale up Not easy to prepare particles	180 nm–50 µm [83]

**Table 2:** SF-based drug delivery systems.

Type of Drug Delivery System	Associated API	Results	References
SF sponges	Erythromycin	Sustained drug release and prolonged antimicrobial activity against Staphilococcus Aureus	[3]
SF films	Horseradish peroxidase (HRP)	Enhanced stability	[36]
	Glucose oxidase (GOx)	Increased enzymatic activity	[91]
	FITC-dextran	Controlled drug release	[95]
	Epirubicin	Controlled drug release	[7]
SF lyogels	Hydrocortisone IgG	Enhanced efficacy Enhanced stability and sustained release	[84]
Insertable SF discs	IgG and HIV inhibitor 5P12-RANTES	Enhanced stability and modified release profile	[27]
SF nanoparticles	Curcumin	Modified release profile and enhanced cellular uptake	[43]
SF microspheres	Horseradish peroxidase (HRP)	Modified the release profile	[96]
SF-coated PCL microspheres	Vancomycin	Modified the release profile	[98]
SF-coated liposomes	Ibuprofen	Enhanced adhesion to human corneal epithelial cells, tunable drug release	[16]
	Emodin	Selective targeting of keloid cells	[100]

**Table 3:** SF -based formulations for gene delivery.

Formulation	Gene	Cell line	Reference
**Recombinant silk–elastin-like polymer hydrogels (SELPs)**	Adenovirus Ad^1^–CMV^2^–LacZ^3^	Head and neck cancer in mice	[114]
	pDNA^4^ (pRL^5^-CMV-luc^6^)	NA	[115]
	Ad–Luc–HSVtk^7^	Head and neck cancer in mice	[116]
**3D porous scaffold**	Adenovirus Ad-BMP7^8^	Human BMSCs	[117]
**Bioengineered silk films**	pDNA (GFP^9^)	Human HEK cells	[105]
**Spermine modified SF**	pDNA and VEGF165–Ang-1^10^	In vivo-rat	[4]
**SF-Coated PEI/DNA Complexes**	pDNA (GFP)	HEK 293 and HCT 116 cells	[110]
**SF layer-by-layer assembled** **microcapsules**	pDNA-Cy5^11^	NIH/3T3 fibroblasts	[118]
**Bioengineered silk–polylysine–ppTG1 nanoparticles**	pDNA	Human HEK and MDA-MB-435 cells	[113]
**Magnetic-SF/polyethyleneimine core-shell nanoparticles**	c-Myc^12^ antisense ODNs^13^	MDA-MB-231 cells	[9]

^1^ Adenovirus; ^2^ cytomegalovirus promoter gene; ^3^ beta galactosidase reporter gene; ^4^ plasmid DNA; ^5^ renilla luciferase; ^6^ luciferase reporter gene; ^7^ herpes simplex virus thymidine kinase gene; ^8^ bone morphogenic protein; ^9^ green fluorescent protein; ^10^ vascular endothelial growth factor and angiopoietin-1; ^11^ fluorescent probe; ^12^ MYC Proto-Oncogene; ^13^ oligodeoxynucleotides.
